# Dihydroberberine alleviates Th17/Treg imbalance in premature ovarian insufficiency mice via inhibiting Rheb/mTOR signaling

**DOI:** 10.1186/s10020-024-00971-z

**Published:** 2024-10-29

**Authors:** Disi Deng, Yeke Wu, Keming Wu, Nan Zeng, Wanjing Li

**Affiliations:** 1 Department of Gynaecology, Hospital of Chengdu University of Traditional Chinese Medicine, No.39-41, Shijiqiao Road, Jinniu District, Chengdu, 610075 Sichuan Province China; 2 State Key Laboratory of Southwestern Chinese Medicine Resources, Chengdu University of Traditional Chinese Medicine, No.1166, Liutai Avenue, Wenjiang District, Chengdu, 611137 Sichuan Province China; 3 Department of Dentistry, Hospital of Chengdu University of Traditional Chinese Medicine, No.1166, No.39, Shijiqiao Road, Jinniu District, Chengdu, 610075 Sichuan Province China; 4 Fujian Maternity and Child Health Hospital College of Clinical Medicine for Obstetrics & Gynecology and Pediatrics, Fujian Medical University, No. 18, Daoshan Road, Fuzhou, 350001 Fujian Province China; 5 College of Clinical Medicine, Chengdu University of Traditional Chinese Medicine, No.1166, Liutai Avenue, Wenjiang District, Chengdu, 611137 Sichuan Province China

**Keywords:** Dihydroberberine, Premature ovarian insufficiency, Th17/Treg immune imbalance, Rheb/mTOR signaling pathway, CD4 + T cell differentiation

## Abstract

**Background:**

Premature ovarian insufficiency (POI) is an immune-related condition. Dihydroberberine (dhBBR) plays a regulatory role in maintaining the T-helper 17 (Th17)/regulatory T (Treg) cell balance. This study aimed to explore the action mechanisms of dhBBR on POI.

**Methods:**

In vivo, female BALB/c mice were used as POI models, treated with dhBBR, or injected with recombinant interleukin (rIL)-17 and anti-CD25 monoclonal antibody. Hematoxylin and eosin staining was used to validate the model and assess the therapeutic effects of dhBBR. mRNA expression levels of cytochrome P450 (Cyp)-*17a1*, *Cyp19a1*, *Cyp11a1*, steroidogenic acute regulatory protein, and luteinizing hormone receptor in mouse ovaries were quantified via quantitative polymerase chain reaction (qPCR). Enzyme-linked immunosorbent assay was used to determine the cytokine and sex hormone levels. Immunohistochemical staining for cleaved-caspase 3 and Ki-67 were performed to assess ovarian cell apoptosis and proliferation. Flow cytometry was used to analyze the Th17/Treg cell balance in the ovary and spleen. In vitro cytotoxicity of dhBBR was measured using the cell counting kit-8 assay. GTP-Ras homolog enriched in brain (Rheb) activity was determined via immunofluorescence assay. Co-immunoprecipitation was performed to assess Rheb activity, Th17 or Treg induction, and binding between Rheb and mammalian target of rapamycin (mTOR) after dhBBR treatment. Flow cytometry and qPCR assays were used to verify the effect of dhBBR on CD4 + cell differentiation. Finally, Rheb/mTOR pathway activation was confirmed via western blotting of proteins, including mTOR, p-mTOR, p70S6K, p-p70S6K, 4E-BP1, and p-4E-BP1.

**Results:**

dhBBR improved the ovarian function in a dose-dependent manner. It also decreased ovarian cell apoptosis and increased cell proliferation. It decreased Th1 and Th17 cell proportions but increased Treg cell proportions in the ovaries and spleens of POI model mice. Cell experiments revealed that dhBBR promoted CD4 + cell differentiation into Treg cells. Co-immunoprecipitation revealed Rheb as the dhBBR target that bound to mTOR. However, MHY1485 restored dhBBR-induced changes in forkhead box P3, IL-10, transforming growth factor-β1, IL-17, IL-22, retinoic acid-related orphan receptor-γt and p-mTOR levels in Th17- and Treg-induced CD4 + cells.

**Conclusion:**

Overall, dhBBR targeted the Rheb/mTOR pathway to promote CD4 + cell differentiation into Treg cells and alleviate POI.

**Supplementary Information:**

The online version contains supplementary material available at 10.1186/s10020-024-00971-z.

## Background

Premature ovarian insufficiency (POI), a condition characterized by impaired ovarian function, primarily manifests as premature menopause in women under 40 years of age (European Society for et al. 2016). It is characterized by amenorrhea for over four months, lack of functional ovarian follicles, elevated serum follicle-stimulating hormone (FSH) levels, and estradiol deficiency. POI affects 0.5–3% of women of reproductive age, and its onset is associated with various factors. Genetic factors, such as chromosomal and genetic abnormalities, account for approximately 10.8% of all POI cases (Rudnicka et al. 2018). Iatrogenic factors, such as cyclophosphamide treatment, increase the risk of POI to 40% (Ishizuka 2021). Additionally, autoimmune factors are responsible for approximately 4–30% of POI cases (Sharif et al. 2019). These reports highlight immunomodulation as a key target for POI treatment.

Ovaries and the immune system are significantly correlated. In 1972, researchers first revealed that newborn female mice exhibit ovarian dysfunction after thymus removal (Sakakura and Nishizuka 1972). POI is associated with autoimmune disorders(Torrealday et al. 2017). Imbalance in the T-helper 17 (Th17)/regulatory T (Treg) cell ratio is common in patients with POI, who mainly exhibit increased Th17 cell proportions and decreased Treg cell proportions. This imbalance in cell ratio is associated with elevated levels of pro-inflammatory cytokines, such as interleukin (IL)-17 and IL-6 (Hu et al. 2022). Some studies have explored the feasibility of using Th17/Treg cells as potential biomarkers and therapeutic targets for POI. POI can be predicted early by detecting the changes in the Th17/Treg cell ratio in the peripheral blood (Shuai et al. 2023). Intraperitoneal injection of CD4 + CD25 + Treg cells alleviates POI in mice (Liu et al. 2020). However, the molecular mechanisms involved in POI remain unclear.

Berberine (BBR; C20H18NO4) is an alkaloid extracted from traditional medicinal plants, primarily the *Berberidaceae* members, including *Coptis chinensis*, *Phellodendron chinense*, and *Berberis thunbergii* (Refaat et al. 2013). BBR exhibits various biological activities, including antibacterial, anti-inflammatory, blood sugar-lowering, lipid-lowering, and antitumor activities (Liu et al. 2019; Hu et al. 2013). It also plays a key role in regulating T cell immunity and differentiation (Yang et al. 2024a, b). BBR activates the AMP-activated protein kinase pathway and increases forkhead box P3 (FOXP3) levels, thereby promoting Treg cell differentiation in chronic colitis mice (Takahara et al. 2019). Dihydroberberine (dhBBR; C20H20NO4) is the reduced form of BBR. Owing to structural differences, dhBBR exhibits higher absorption and bioavailability in animals than BBR (Feng et al. 2015). Although no studies have investigated the effects of dhBBR on POI, some studies have explored the effects of BBR on POI and polycystic ovary syndrome. For example, *Peng Y*, et al. reported that BBR alleviates oxidative stress and inflammatory responses in POI model mice (Peng et al. 2023). Another study demonstrated that the effects of BBR therapy on polycystic ovary syndrome are related to the inhibition of ovarian apoptosis (Peng et al. 2023; Shen et al. 2021). Therefore, we hypothesized that dhBBR is a target drug for POI; however, its effects remain unclear.

Through molecular docking predictions, we previously found that dhBBR strongly binds with the Ras homolog enriched in the brain (Rheb). Rheb protein is a small GTPase that is a major activator of the mammalian target of rapamycin complex 1 (mTORC1) (Shimobayashi and Hall 2016). When Rheb binds to GTP, it directly interacts with and activates mTORC1, thereby promoting protein synthesis, lipid synthesis, and cell growth. Currently, Rheb/mTOR signaling has been mainly explored in cancer (Bao et al. 2019). However, its effects on the Th17/Treg cell balance and POI remain unclear. Therefore, in this study, we aimed to explore whether dhBBR targets Rheb/mTOR to restore the Th17/Treg cell balance using POI model mice.

## Methods

### Molecular docking prediction

mTOR structure was determined using the Protein Data Bank (https://www.rcsb.org). The Traditional Chinese Medicine Systems Pharmacology database was used to obtain dhBBR structure data file. Docking results of mTOR/dhBBR were visualized using the Pymol 2.4.0 software (Schrödinger, USA).

### POI mouse modeling and drug treatment

Sixty female BALB/c mice (7–8 weeks old; 18–22 g) were used in this study (Chengdu Dashuo Biotechnology Co., Ltd, China). A mouse model of POI was established via subcutaneous multisite injection of mouse zona pellucida glycoprotein 3 (pZP3) (Chinese Peptide Co., Ltd, China). Briefly, pZP3 lyophilized powder was dissolved in double-distilled water and mixed with an equal volume of Freund’s complete adjuvant (Sigma, USA) to achieve a working concentration of 50 nmol/L. Each mouse received 0.1 mL of pZP3 via subcutaneous injection at multiple sites, including the toes and abdomen. Two weeks after initial immunization, the same method was used for second subcutaneous injection of 0.1 mL pZP3 per mouse with Freund’s incomplete adjuvant. Vaginal cytology smears were performed 5–6 weeks after first immunization. Two consecutive disrupted estrous cycles and significantly increased anti-zona pellucida antibody levels confirmed successful establishment of the POI model. A normal estrous cycle involved a regular series of pre-estrus, estrus, and post-estrus phases separated by four or five days. Any pattern outside this regular cycle was considered abnormal (Carolino et al. 2019). This study was approved by the Animal Ethics Committee of Fujian Medical University (No. IACUC FJMU 2023 − 0214).

In the 8th week after initial immunization, sixty mice were randomly divided into the following groups described below. Group 1: Control, POI, POI + L-dhBBR, POI + M-dhBBR, and POI + H-dhBBR; mice in the POI group received daily oral administration of 12.5, 25, and 50 mg/kg dhBBR. Group 2: Control, POI, POI + dhBBR, POI + dhBBR + recombinant IL-17 (rIL-17), and POI + dhBBR + anti-CD25 monoclonal antibody (aCD25mAb); mice in the POI group received daily oral administration of 50 mg/kg dhBBR. Additionally, treatment groups received intraperitoneal injections of 100 ng/mice IgG, 100 ng/mice rIL-17, and 250 µg/mice aCD25mAb thrice a week. Treatment continued for four weeks. At the end of the experiment, the mice were anesthetized with isoflurane, their blood was collected, and they were euthanized. Their spleens and ovaries were harvested for analysis.

### CD4 + T cell differentiation and drug treatment

Primary CD4 + T cells were purchased from Biobw (Bio-53558; Changsha, China) and cultured in the Roswell Park Memorial Institute-1640 medium (R8758, Sigma-Aldrich, USA) supplemented with 10% fetal bovine serum, 100 U/mL penicillin, 100 µg/mL streptomycin, 2 mM L-glutamine, and 50 µM 2-mercaptoethanol. After reaching the logarithmic growth phase, CD4 + T cells were differentiated into Treg and Th17 cells under conditions described below. For the Treg cell differentiation, CD4 + T cells were mixed with the anti-CD3 (2 µg/mL), anti-CD28 (1 µg/mL), anti-IL-2 (10 ng/mL), and anti-transforming growth factor (TGF)-β (8 ng/mL) antibodies and cultured for three days. For Th17 cell differentiation, CD4 + T cells were mixed with the anti-CD3 (2 µg/mL), anti-CD28 (1 µg/mL), anti-IL-23 (20 ng/mL), anti-IL-6 (20 ng/mL), anti-TGF-β (2 ng/mL), anti-interferon (IFN)-γ (5 µg/mL), and anti-IL-4 (5 µg/mL) antibodies and cultured for four days.

After differentiation, the cells were treated with different doses of dhBBR or MHY1485 (mTOR agonist; 5 μm). The cells were divided into groups as described below. Group 1: Control, Treg-induced, Treg-induced + L-dhBBR (0.25 µM), Treg-induced + M-dhBBR (0.5 µM), and Treg-induced + H-dhBBR (1 µM). Group 2: Control, Th17-induced, Th17-induced + L-dhBBR (0.25 µM), Th17-induced + M-dhBBR (0.5 µM), and Th17-induced + H-dhBBR (1 µM). Group 3: Control, dhBBR (1 µM), Treg-induced, Treg-induced + dhBBR, Th17-induced, and Th17-induced + dhBBR. Group 4: Th17-induced, Th17-induced + dhBBR, and Th17-induced + dhBBR + mTOR agonist. Group 5: Treg-induced, Treg-induced + dhBBR, and Treg-induced + dhBBR + mTOR agonist.

### Hematoxylin and eosin (H&E) and immunohistochemical (IHC) staining

Ovaries and spleens were removed from mice and fixed with 4% paraformaldehyde (158127, Sigma-Aldrich, USA), dehydrated, and cut in 5-um sections. Ovarian samples were used for H&E and IHC staining of cleaved-caspase 3 and Ki-67. Spleen samples were subjected to GTP-Rheb IHC staining. hematoxylin (ST2067, Beyotime, China) and eosin (YE2080, Hefei Bomei Biotechnology Co., Ltd, China) were used to perform H&E staining, according to the manufacturer’s instructions. For IHC staining, tissue sections were incubated with 3% H_2_O_2_ for 10 min at room temperature. After blocking with bovine serum, the samples were incubated with primary antibodies against cleaved-caspase 3 (1:100; GB11532; ServicebioChina), Ki-67 (1:400; Cat. HA721115; HUABIOChina ), and GTP-Rheb (1:100; Cat. 15924-1-AP; ProteintechUSA) at 4 °C overnight. After washing thrice with phosphate-buffered saline (PBS), the samples were incubated with the secondary antibodies (1:100; Cat. GB23303; ServicebioChina ) at 37 °C for 30 min. Finally, changes were visualized using the DAB kit (1:20; Cat. ZLI-9018; ZsbioChina). Images were analyzed using an image analysis system (Halo 101-WL-HALO-1; Indica Labs) and Digital Trinocular Camera Microscope.

### Enzyme-Linked Immunosorbent Assay (ELISA)

Mouse aZP (ZC-38389; ZCIBIO, China), mouse IFN-γ (ZC-37905; ZCIBIO), mouse TGF-β1 (ZC-39043; ZCIBIOChina ), mouse IL-17 A (ZC-37971; ZCIBIO), mouse tumor necrosis factor (TNF)-α (ZC-39024; ZCIBIO), mouse IL-10 (ZC-37962; ZCIBIOChina ), Mouse E2 (ZC-38046; ZCIBIO China), mouse FSH (ZC-38059; ZCIBIOChina), mouse IL-23 (ZC-37979; ZCIBIOChina), mouse IL-35 (ZC-37985, ZCIBIOChina ), and mouse anti-Mullerian hormone (AMH; ZC-38503; ZCIBIO, China ) ELISA kits were used to determine the protein expression levels in the serum samples of each mouse group. SpectraMAX Plus384 enzyme marker (Molecular Devices, Santa Clara, CA) was used to measure the optical density at 450 nm.

### Quantitative Polymerase Chain Reaction (qPCR) assay

Next, mRNA levels of cytochrome P450 (*Cyp*)-*17a1*, *Cyp19a1*, *Cyp11a1*, steroidogenic acute regulatory protein (*Star*), and luteinizing hormone receptor (Lhr) in mouse ovaries and *Foxp3*, *TGF-β1*, *IL-10*, retinoic acid-related orphan receptor (*ROR*)-*γt*, *IL-17*, *IL-22*, and *Rheb* in CD4 + T cells were quantified via qPCR. Total RNAs in ovarian tissues and CD4 + T cells were extracted using the Molpure Cell/Tissue Total RNA Kit (19221ES50; YEASEN, Shanghai, China), and reverse-transcribed using the PrimeScript RT reagent Kit (RR047A; Takara, Japan). TB Green Premix Ex Taq II (RR820A; Takara) was used to perform qPCR, and QuantStudio Design & Analysis SE Software (Thermo Fisher, USA) was used to analyze the CT value; fold change was normalized using the 2^−△△CT^ method. All primers are listed in Table 1. PCR conditions were set as follows: 95 °C for 30 s, followed by 45 cycles at 95 °C for 5 s, 55 °C for 30 s, and 72 °C for 30 s.

**Table 1:** Primers in this study

Gene name	Forward primer 5’-3’	Reverse primer 5’-3’
**β-actin**	CTACCTCATGAAGATCCTGACC	CACAGCTTCTCTTTGATGTCAC
**Cyp11a1**	AGTATTATCAGAGGCCCATTGG	AACATCTGGTAGACAGCATTGA
**Cyp17a1**	GAGGTGAAGAGGAAGATCCAAA	ATACGAAGCACTTCTCGGATAG
**Cyp19a1**	TCA TGA AGC ACA GTC ACT ACA T	AAA CTT CCA CCA TTC GAA CAA C
**Lhr**	TTTCTCTTGCTGAGCAGATTTG	TAGGTTGTTGACAGTGCACTAT
**Star**	TCAACTGGAAGCAACACTCTAT	ATCTTACTTAGCACTTCGTCCC

### Cell Counting Kit (CCK)-8 assay

Log-phase CD4 + T cells were collected and washed with PBS. CD4 + T cells (5 × 10^3^) were seeded in a 96-well-plate and cultured at 37 °C and 5% CO_2_. Then, dhBBR (0, 0.01, 0.1, 1, 10, and 100 µM) was added for 24 h, followed by the addition of CCK-8 working solution (no. BS350B; Biosharp, China) for 2 h at 37 °C and 5% CO_2_. Optical density was measured at 450 nm.

### Flow Cytometry (FCM) assay

Ovarian and spleen tissues were washed with PBS and ground. The cell suspension was collected, filtered through a 200-mesh cell sieve, centrifuged, and washed. Then, log phase CD4 + T cells were collected, and cultured at 2 × 10^5^ cells/well with drugs for Treg or Th17 induction. After mixing with the cell activation cocktail, the cells were cultured for 4 h at 37 °C and centrifuged at 350 × *g*, and the supernatant was discarded. Cells mixed with 100 µL PBS were incubated with anti-CD4 and anti-CD25 antibodies for 30 min at 4 °C in the dark. Then, 500 µL 1× True Nuclear Fixation Concentrate was added to each well and incubated for 50 min. After washing, FOXP3 and IL-17 A labeling was performed for 30 min at 4 °C. Finally, data were analyzed using the Flow Analyzer (Cytoflex; Beckman, USA).

### Western Blotting (WB) assay

Spleens from each POI mouse group were ground, and CD4 + T cells were collected to assess the Rheb/mTOR pathway. Tissues and cells were lysed using the radioimmunoprecipitation assay lysis buffer. Protein concentration was determined using the BCA Protein Assay Kit (P0009; Beyotime, China). Sodium dodecyl sulfate-polyacrylamide gel electrophoresis was conducted to separate the protein sample (20 µg), which was transferred to the PDGF membrane. After incubating with primary antibodies against TOR (1:5000; 66888-1-Ig; Proteintech, USA), p-mTORSer2448 (1:2000; 67778-1-Ig; Proteintech, USA), p70S6K (1:1000; A2190; Abclonal, China), p-p70S6K (1:1000; AP0502; Abclonal), 4E-BP1 (1:1000; A24691; Abclonal, China), p-4E-BP1 (1:2000; AP1363; Abclonal), Rheb (1:1000; 13879; Cell Signaling Technology), RORγt (1:1000; bs-23109R; Bioss, China), and β-actin (1:5000; AC026; Abclonal, China) 4 °C overnight, the membrane was washed with PBS and incubated with HRP goat anti-mouse IgG (H + L) (1:5000; AS003; Abcam) and goat anti-aabbit IgG (H + L) HRP (1:5000; S0001; Affbiotech, China) secondary antibodies at room temperature for 2 h. Densitometric analysis was performed using a Gel Imaging System (Analytik Jena US LLC).

### Co-Immunoprecipitation (Co-IP) assay

Co-IP was performed to determine Rheb protein enrichment in CD4 + T cells and binding between Rheb and mTOR in 293T cells. For CD4 + T cells, IP was performed using antibody-conjugated agarose beads against GTPγS and GDP. The cells were divided into seven groups: GDP, GTPγS, GTPγS + 1 µM dhBBR, GTPγS + Treg-induced, GTPγS + Treg-induced + dhBBR, GTPγS + Th17-induced, and GTPγS + Th17-induced + dhBBR groups. Then, 293T cells were transfected with Myc-tagged Rheb expression plasmid (Myc-Rheb) for 24 h and co-incubated with 0.25, 0.5, and 1 µM dhBBR for 24 h. CD4 + T or 293T cells (CL-0005; Wuhan) from each group were collected and washed thrice. Then, 10^6^ cells from each group were mixed with the 100 µL IP lysis buffer (including protease inhibitor) to lyse the cells on ice for 30 min. A co-IP kit (PK10007; Proteintech) was used for analysis. Subseqeuntly, 3 mg whole-cell lysates was incubated with specific antibodies against Rheb (13879; Cell Signaling Technology) and rabbit IgG (A7016; Beyotime) overnight at 4 °C. Protein A Sepharose bead slurry was used to precipitate the immune complexes, and spin columns were used to elute the products. Then, products (10 µL) were subjected to sodium dodecyl sulfate-polyacrylamide gel electrophoresis to separate the proteins. WB was performed using HRP-conjugated protein A (PK10007; Proteintech), anti-mTOR (66888-1-Ig; Proteintech), and anti-Myc (ab32; Abcam) antibodies, according to the manufacturers’ protocol.

### Immunofluorescence (IF) assay

IF was performed to determine GTP-Rheb levels in CD4 + T cells treated with dhBBR with Treg or Th17 induction. After 20-min serum bloking, the cells in each group were incubated with the GTP-Rheb antibody (1:100; 15924-1-AP; Proteintech) overnight at 4 °C. The sections were washed thrice and incubated with the FITC-labeled goat anti-rabbit antibody (1:100; GB22303; Servicebio), followed by incubation with 4’,6-diamidino-2-phenylindole (G1012; Servicebio) for 10 min. Digital Scanning Viewing Software (ImageJ; National Institutes of Health) and Data Image Analysis System (OlyVIA; OLYMPUS) were used to analyze GTP-Rheb expression.

### Statistical analyses

GraphPad Prism 8.0 (GraphPad Software, Inc. USA) was used to analyze the data. All data are represented as the mean ± standard deviation. Statistical significance was set at *P* < 0.05. One-way analysis of variance, followed by Tukey’s multiple comparisons test was used to compare multiple groups, whereas an unpaired two-tailed Student’s *t*-test was used to compare two groups.

## Results

### Effects of dhBBR on the hormone levels and follicular development in POI model mice

**Fig. 1:**
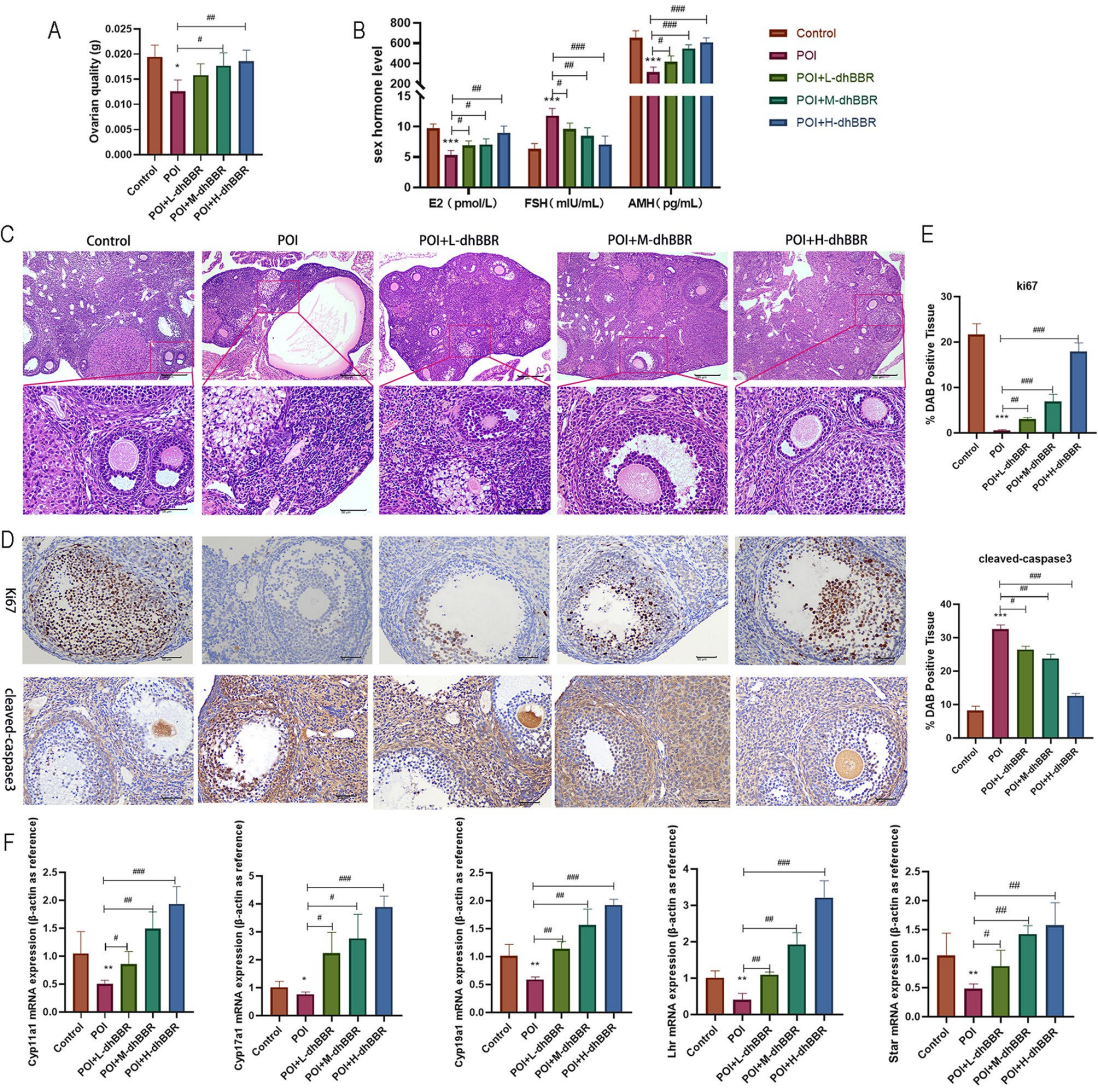
The therapeutic effect of dhBBR on POI mice. POI mice were fed with 12.5, 25, and 50 mg/kg dhBBR, respectively. **A**: Ovarian quality of each group. **B**: Sex hormone levels including E2, FSH, and AMH were detected by ELISA. **C**: The ovarian pathologies were observed by HE staining (100x: bar = 200 μm; 400x: bar = 50 μm). **D-E**: IHC staining was performed to determine proteins Ki67 and cleaved-caspase3 expression (bar = 50 μm). **F**: qPCR was used to quantify mRNA expressions of Cyp17al, Cyp19al, Cyp11al, Star, and Lhr. Vs. the control group, ^*^p < 0.05, ^**^p < 0.01, ^***^p < 0.001; vs. the POI group, ^#^*p* < 0.05, ^##^*p* < 0.01, ^###^*p* < 0.001

POI model mice were administered 12.5 mg/kg (low dose), 25 mg/kg (medium dose), or 50 mg/kg (high dose) dhBBR. As shown in Fig. 1A, ovarian function was decreased in the POI model but restored by high-dose dhBBR. Compared to the control group, POI group exhibited lower E2 and AMH levels and higher FSH levels. Compared to the POI group, dhBBR treatment groups exhibited higher E2 and AMH levels and lower FSH levels (Fig. 1B). Subsequently, H&E staining was performed to observe the ovarian pathology. As shown in Fig. 1C, POI group exhibited decreased number of ovarian follicles, cystic follicular dilatation, and necrosis of follicular cells compared to the control group; however, these effects were alleviated by dhBBR treatment in a dose-dependent manner. Ki67 and cleaved-caspase 3 protein levels showed opposite trends in the POI and dhBBR treatment groups; dhBBR significantly reversed the POI-induced decrease in Ki67 levels (Fig. 1D–E). Furthermore, mRNAs levels of *Cyp17a1*, *Cyp19a1*, *Cyp11a1*, *Star*, and *Lhr* were measured via qPCR. As shown in Fig. 1F, levels of all these genes were downregulated in the POI group; however, this effect was reversed by dhBBR, with the highest dose showing the most potent effect.

### Immunomodulatory effects of dhBBR on the spleen of POI model mice via the Rheb/mTOR pathway

**Fig. 2:**
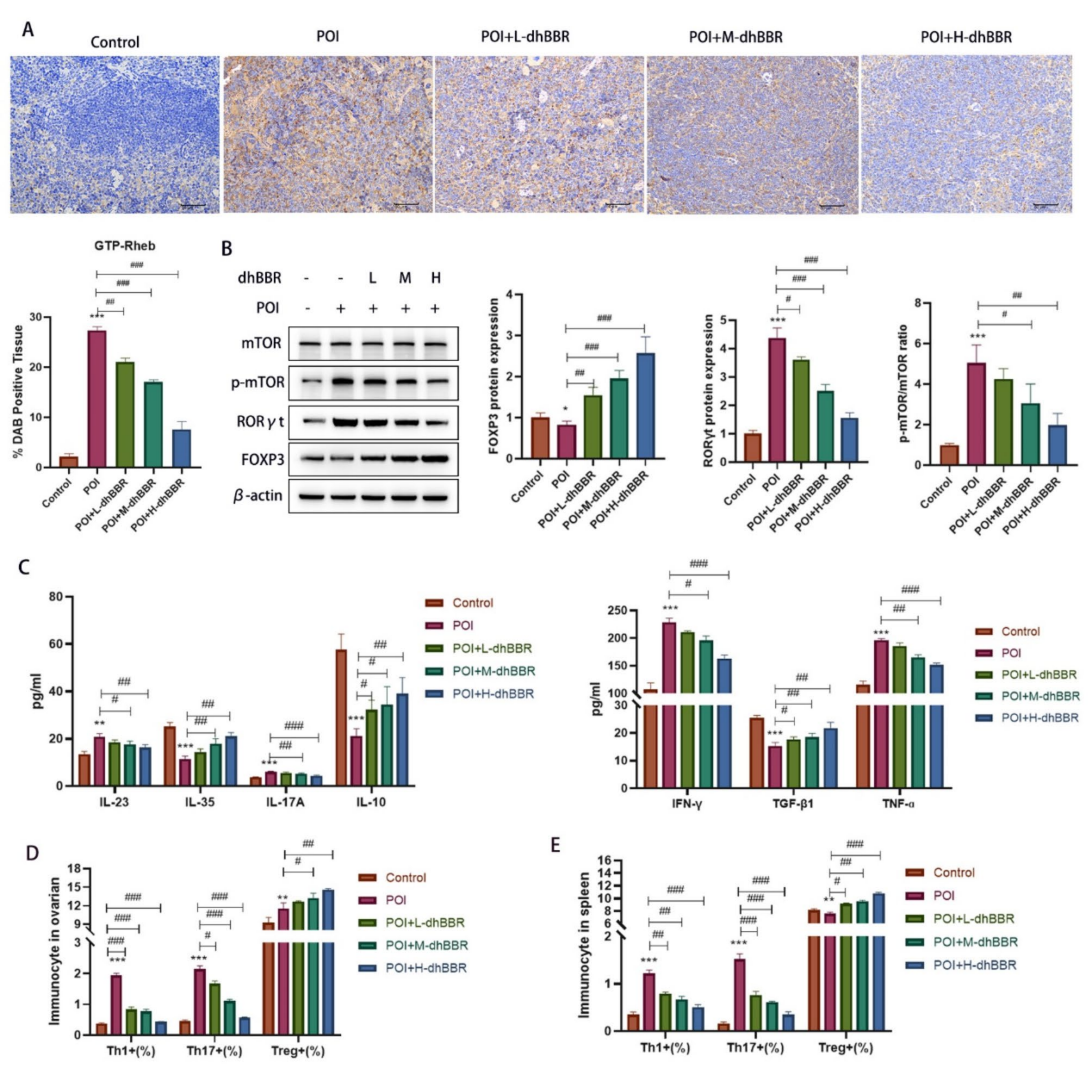
The immunomodulatory effect of dhBBR on Th17/Treg balance in POI mice may target to the Rheb/mTOR signaling pathway. POI mice were fed with 12.5, 25, and 50 mg/kg dhBBR, respectively. **A**: IHC staining was performed to determine GTP-Rheb expression (bar = 50 μm). **B**: Proteins including mTOR, p-mTOR, RORγt, and Foxp3 were detected by WB. **C**: Cytokines, such as IFN-γ, IL-17 A, IL-23, TNF-ɑ, TGF-β1, IL-10, and IL-35 were detected by ELISA. **D**: Flow cytometry was used to quantify the percentage of Th1, Th17, and Tregs in ovarian mice. **E**: Flow cytometry was used to quantify the percentage of Th1, Th17, and Tregs in the spleen of mice. Vs. the control group, ^*^p < 0.05, ^**^p < 0.01, ^***^p < 0.001; vs. the POI group, ^#^*p* < 0.05, ^##^*p* < 0.01, ^###^*p* < 0.001

Next, we explored whether the effect of dhBBR is related to the Rheb/mTOR pathway and Th17/Treg cell balance using the POI model mice. Several genes and proteins were identified. GTP-Rheb expression in the spleen was assessed via IHC staining (Fig. 2A). dhBBR decreased the POI-induced increase in GTP-Rheb levels in a dose-dependent manner. WB was also performed to quantify the mTOR, p-mTOR, RORγt, and Foxp3 levels. As shown in Fig. 2B, Foxp3 levels were decreased in the POI group but increased by dhBBR treatment; however, the opposite trend was observed in RORγt levels and p-mTOR/mTOR ratio. ELISA was used to evaluate the serum cytokine levels. As shown in Fig. 2C, IFN-γ, IL-17 A, IL-23, and TNF-α levels were increased in the POI group but decreased by dhBBR treatment. In contrast, TGF-β1, IL-10, and IL-35 levels were decreased in the POI group but increased by dhBBR treatment. FCM was used to quantify the Th17/Treg cell balance in the ovaries (Fig. 2D and S1) and spleens (Fig. 2E and S1) of POI model mice. In the ovary, Th1, Th17, and Treg proportions were increased in the POI group, whereas Th1 and Th17 proportions were decreased and Treg proportions were significantly increased in the dhBBR treatment groups. In the spleen, dhBBR reversed the POI-induced increase in Th1 and Th17 proportions and decrease in Treg proportions.

### dhBBR improves the ovarian function in POI model mice by regulating the Treg/Th17 cell balance

**Fig. 3:**
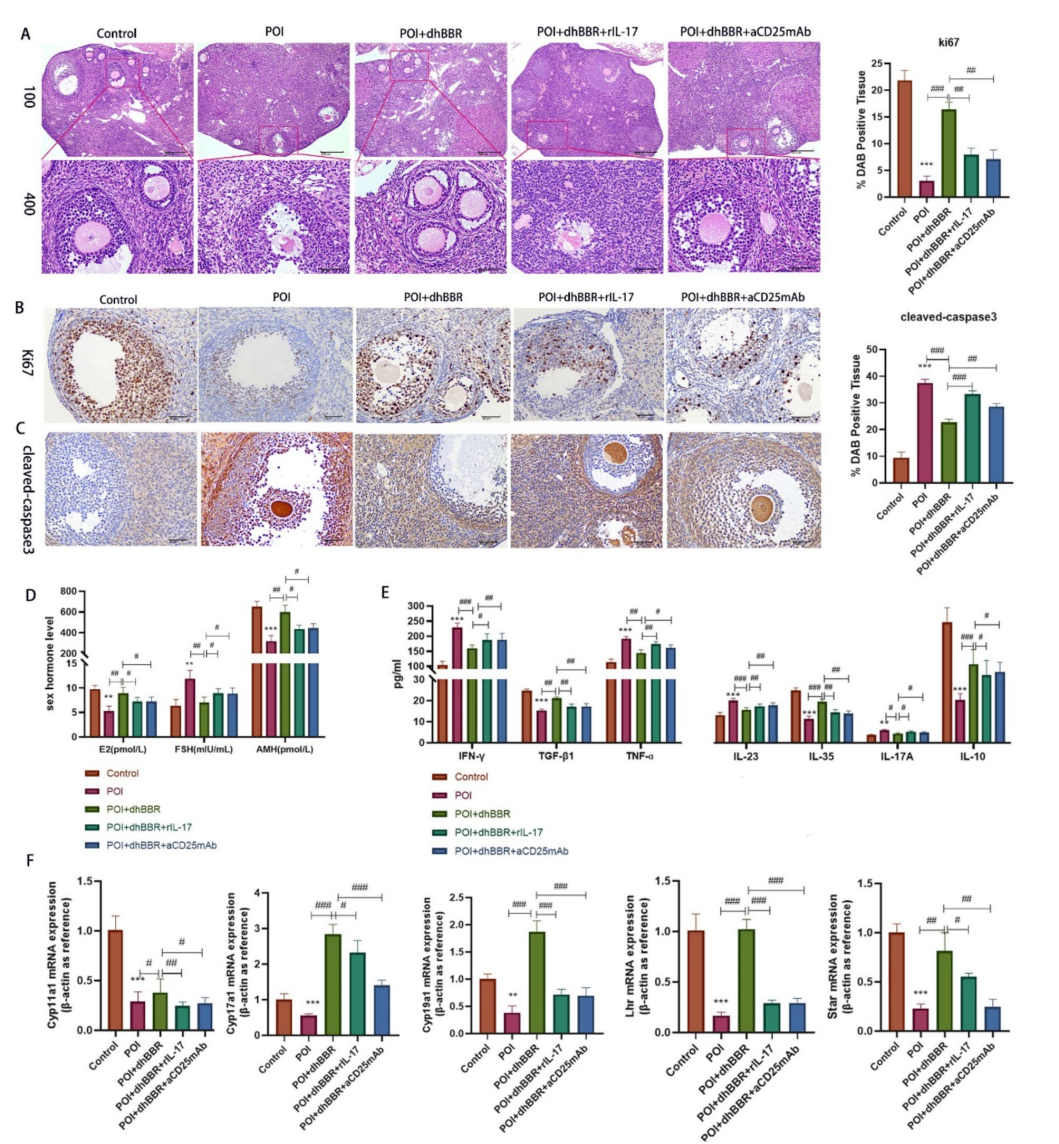
dhBBR improves ovarian function in POI mice by regulating Treg/Th17 cells. POI mice were treated with 1 μM dhBBR, 100 ng rIL-17, and 250 µg aCD25mAb, respectively. **A**: The pathologies of ovarian was observed by HE staining (100x: bar = 200 μm; 400x: bar = 50 μm). **B-C**: IHC staining was performed to determine proteins Ki67 and cleaved-caspase3 expression (bar = 50 μm). **D**: sex hormone levels including E2, FSH, and AMH were detected by ELISA. **E**: cytokines, such as IFN-γ, IL-17 A, IL-23, TNF-ɑ, TGF-β1, IL-10, and IL-35 were detected by ELISA. **F**: qPCR was used to quantify mRNA expressions of Cyp17al, Cyp19al, Cyp11al, Star, and Lhr. Vs. the control group, ^*^p < 0.05, ^**^p < 0.01, ^***^p < 0.001; vs. the POI + dhBBR group, ^#^*p* < 0.05, ^##^*p* < 0.01, ^###^*p* < 0.001

To further investigate the relationship between dhBBR treatment and Treg/Th17 balance in POI, we used rIL-17 to promote Th17 proliferation and aCD25mAb to inhibit Treg activation in dhBBR-treated POI model mice. Figure 3A shows that, compared to the POI + dhBBR group, POI + dhBBR + rIL-17 and POI + dhBBR + aCD25mAb groups exhibited decreased follicle numbers and granulosa cell necrosis. This result suggests that dhBBR targets T-cells to promote Treg activation and proliferation, thereby alleviating POI. IHC revealed that rIL-17 and aCD25mAb mAb reversed dhBBR-induced decreased cleaved-caspase 3 levels (Fig. 3B) and increased ki67 levels (Fig. 3C). Figure 3D shows that rIL-17 and aCD25mAb mAb rescued dhBBR-induced increased E2 and AMH levels and decreased FSH levels in POI model mice. Serum levels of IFN-γ, IL-17 A, IL-23, TNF-α, TGF-β1, IL-10, and IL-35 are shown in Fig. 3E. rIL-17 and aCD25mAb treatment reduced the IFN-γ, IL-17 A, IL-23, and TNF-α levels and increased the TGF-β1, IL-10, and IL-35 levels in POI + dhBBR group. Finally, qPCR was used to quantify the expression of genes related to ovarian function in POI model mice (Fig. 3F). *Cyp17a1*, *Cyp19a1*, *Cyp11a1*, *Star*, and *Lhr* levels were significantly downregulated by rIL-17 and aCD25mAb in the POI + dhBBR group.

### Effect of dhBBR on CD4 + T cell differentiation into Th17 and Treg cells

**Fig. 4:**
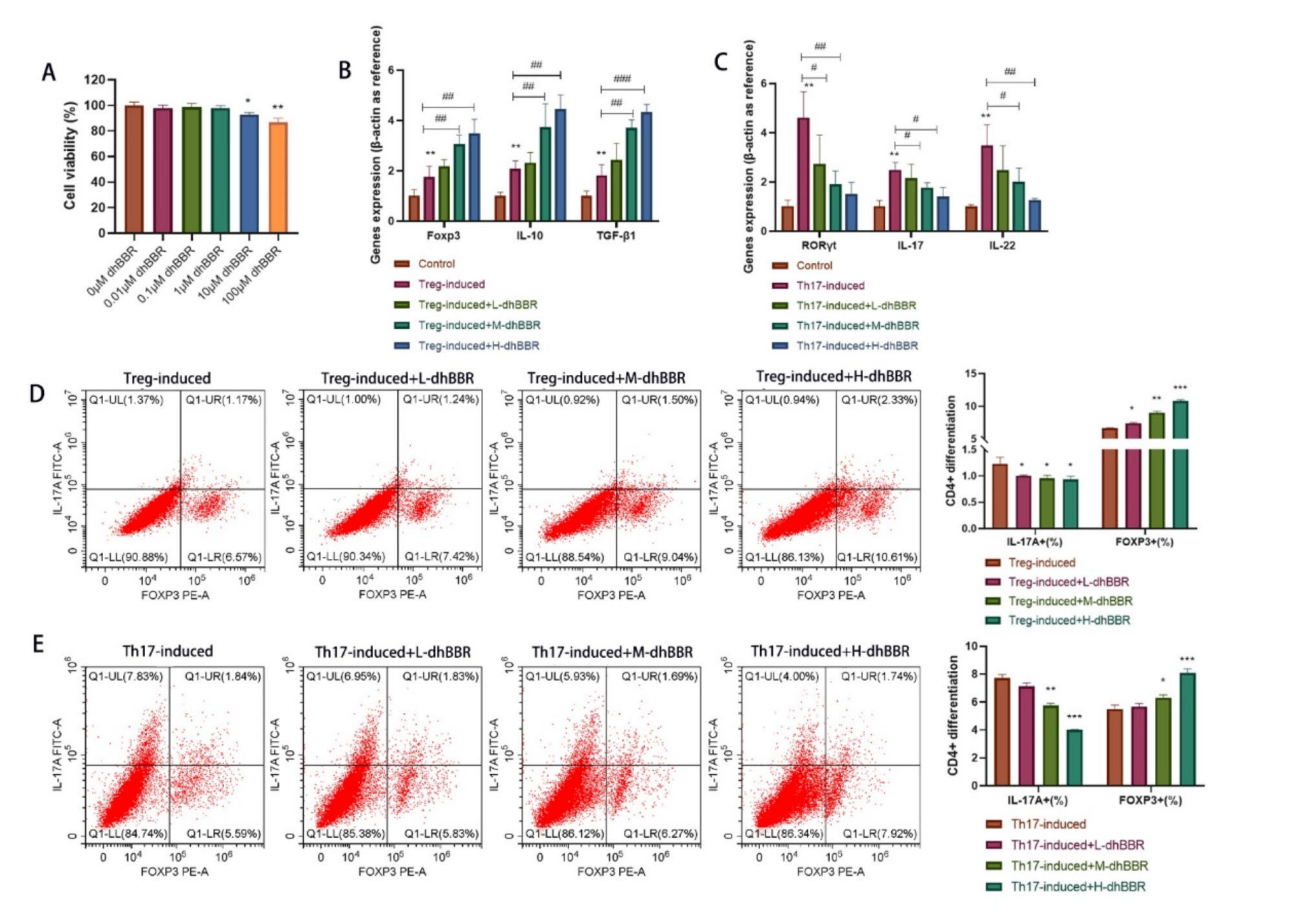
The effect of dhBBR on differentiating CD4 + T cells into Th17 and Treg cells. **A**: CCK-8 was performed to check the CD4 + cells viability under 0.01, 0.1, 1, 10, and 100 μM dhBBR treatment. **B**: qPCR was performed to quantify gene expressions of Foxp3, IL-10, and TGF-β1. **C**: qPCR was performed to quantify gene expressions of IL-17, IL-22, and RORγt. **D**: Tregs and Th17 cells were quantified by flow cytometry when Treg-induced CD4 + cells were treated by 0.25, 0.5, and 1µM dhBBR. **E**: Tregs and Th17 cells were quantified by flow cytometry when Th17-induced CD4 + cells were treated by 0.25, 0.5, and 1µM dhBBR

Our previous study on animal models revealed that the therapeutic effects of dhBBR are related to the Treg/Th17 cell balance. In this study, we induced the differentiation of CD4 + T cells into Treg and Th17 cells and treated them with the highest and non-toxic doses of dhBBR. As shown in Fig. 4A, 1 µM dhBBR was determined as the optimal dose. As shown in Fig. 4B, Foxp3, IL-10, and TGF-β1 levels were increased in the Treg-induced group, which were further increased by dhBBR treatment. As shown in Fig. 4C, IL-17, IL-22, and RORγt levels were increased in the Treg-induced group but decreased by dhBBR treatment. FCM was used to examine the Foxp3+ (%) and IL-17 A+ (%) cells under Th17 (Fig. 4D) and Treg (Fig. 4E) conditions and dhBBR treatment. Foxp3 + cells (%) were increased but IL-17 A + cells (%) were decreased by dhBBR treatment under both Treg and Th17 induction. This result suggests that dhBBR promotes Treg differentiation of CD4 + T cells. Notably, effects of dhBBR were dose-dependent.

### Effect of dhBBR on mTOR/Rheb activation in Treg and Th17 cells

**Fig. 5:**
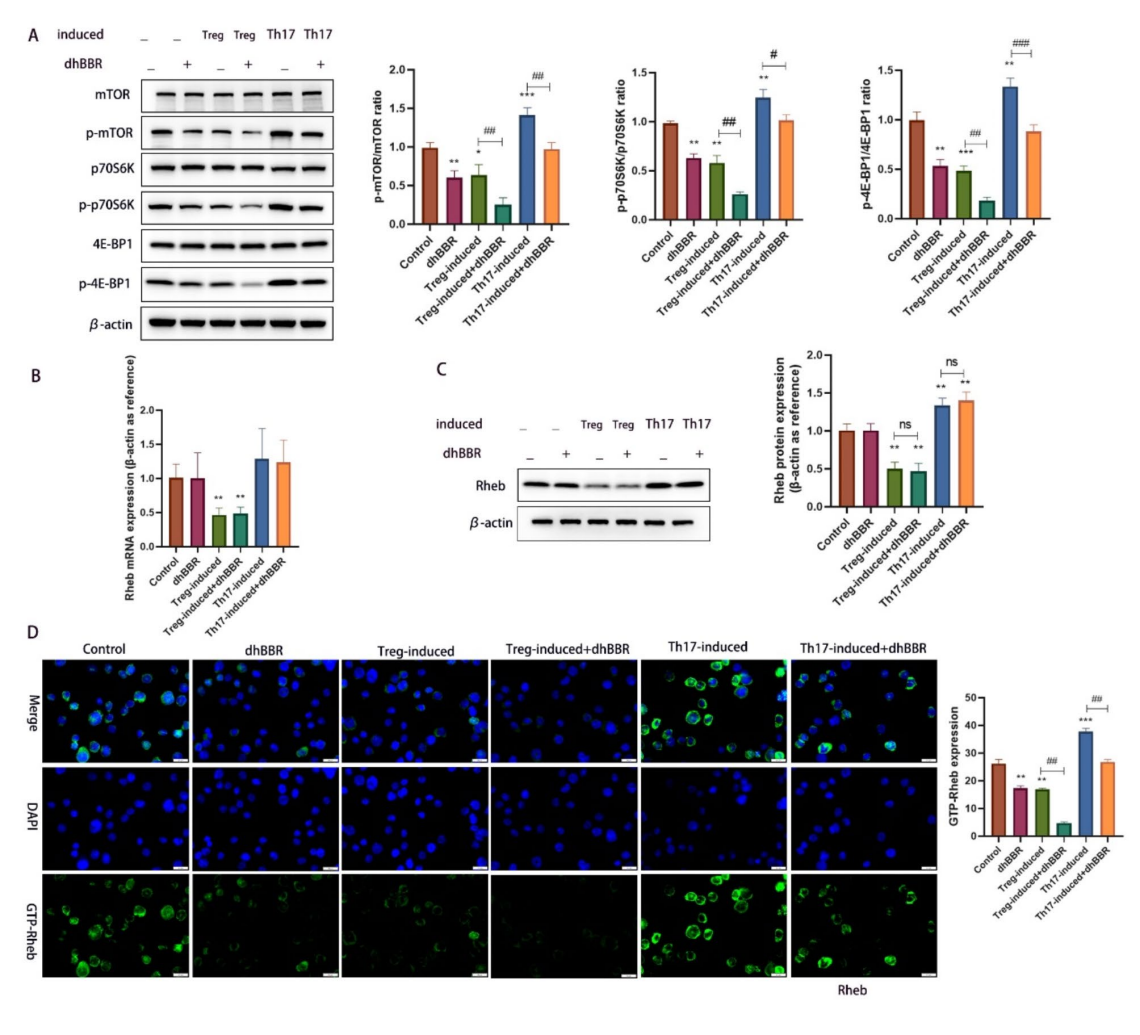
dhBBR targets mTOR/Rheb to impact the CD4 + T cell differentiation. **A**: proteins including mTOR, p-mTOR, p70S6K, p-p70S6K, 4E-BP1, and p-4E-BP1 were detected by WB. **B-C**: the mRNA and protein expression of Rheb was quantified by qPCR and WB. **D**: IF was used to determine the GTP-Rheb activity in CD4 + cells under different treatments. Vs. the control group, ^*^p < 0.05, ^**^p < 0.01, ^***^p < 0.001; vs. the Treg-induced group or Th17-induced group,  ^#^p <0.05,  ^##^*p* < 0.01, ^###^*p* < 0.001

CD4 + cells were induced to differentiate into Treg and Th17 cells and treated with dhBBR. We previously suggested that the therapeutic effects of dhBBR against POI are related to the Rheb/mTOR pathway. To verify this, we determined the protein levels of mTOR, p-mTOR, p70S6K, p-p70S6K, 4E-BP1, and p-4E-BP1. As shown in Fig. 5A, phosphorylation of mTOR, p70S6K, and 4E-BP1 was inhibited by Treg induction but promoted by Th17 induction. However, dhBBR treatment reversed these effects. Rheb levels were quantified using qPCR and WB. As shown in Fig. 5B and **C**, dhBBR treatment had no obvious effect on Rheb levels, whereas Treg induction decreased and Th17 induction increased the Rheb levels.

### Co-IP analysis of the effect of dhBBR on the binding of exogenous Rheb and mTOR

**Fig. 6:**
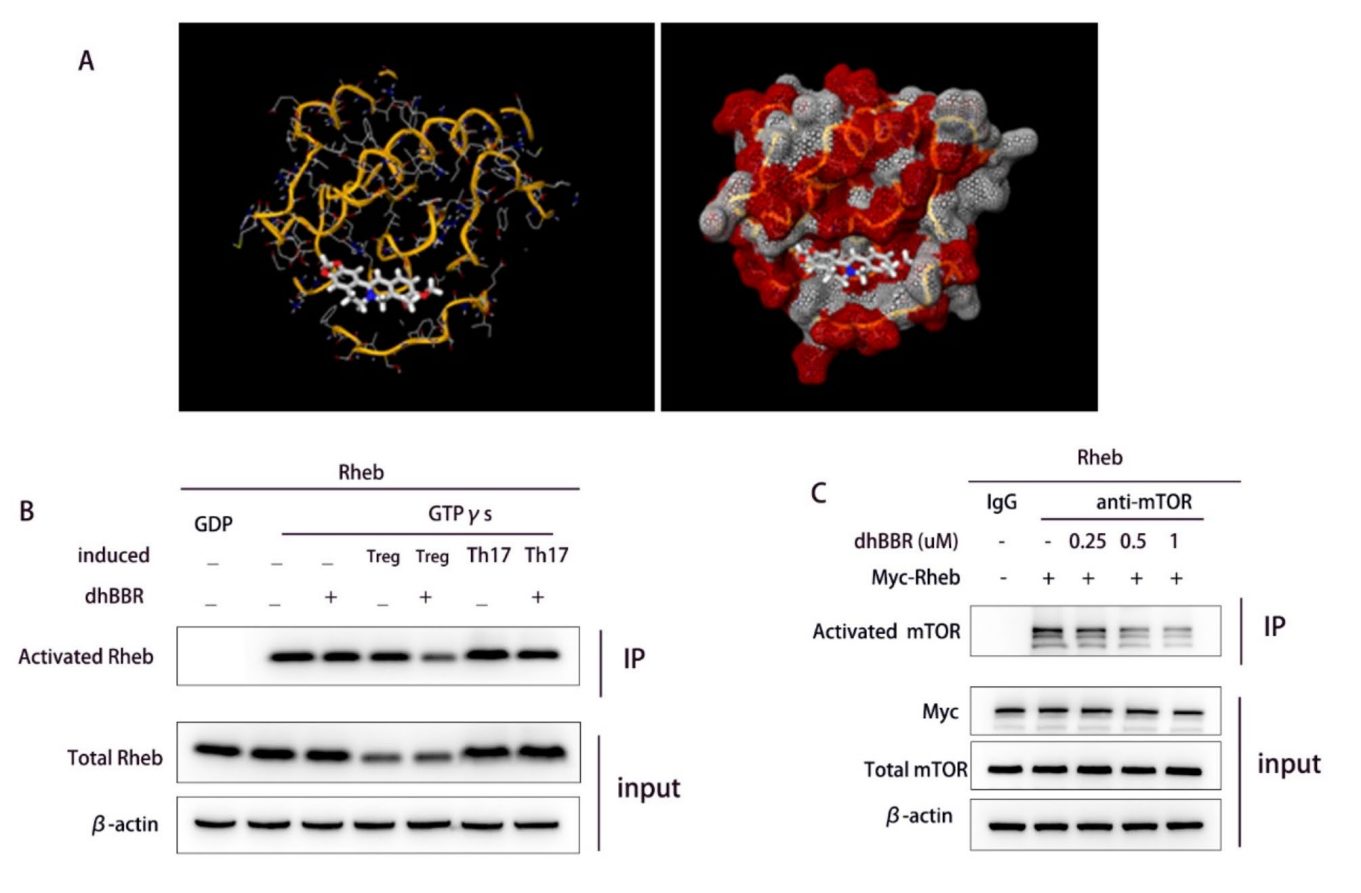
dhBBR targets Rheb. **A**: Molecular docking results for dhBBR and Rheb **B**: The activated Rheb was measured by Co-IP. C: Co-IP verified the binding relationship between Rheb and mTOR

IF was used to determine GTP-Rheb activity in CD4 + cells. As shown in Fig. 5D, dhBBR treatment inhibited GTP-Rheb activity in both Treg- and Th17-induced CD4 + cells. Next, molecular docking predicted Rheb as a target of dhBBR (Fig. 6A**).** We performed Co-IP assays to investigate the effect of dhBBR on Rheb activity during Th17 and Treg differentiation (Fig. 6B). The results were consistent with the IF findings. Co-IP assay was also used to examine the interaction between Rheb and mTOR. As shown in Fig. 6C, Rheb bound to mTOR, and dhBBR inhibited this binding.

### Role of mTOR pathway in dhBBR-mediated CD4 + T cell differentiation into Treg cells

**Fig. 7:**
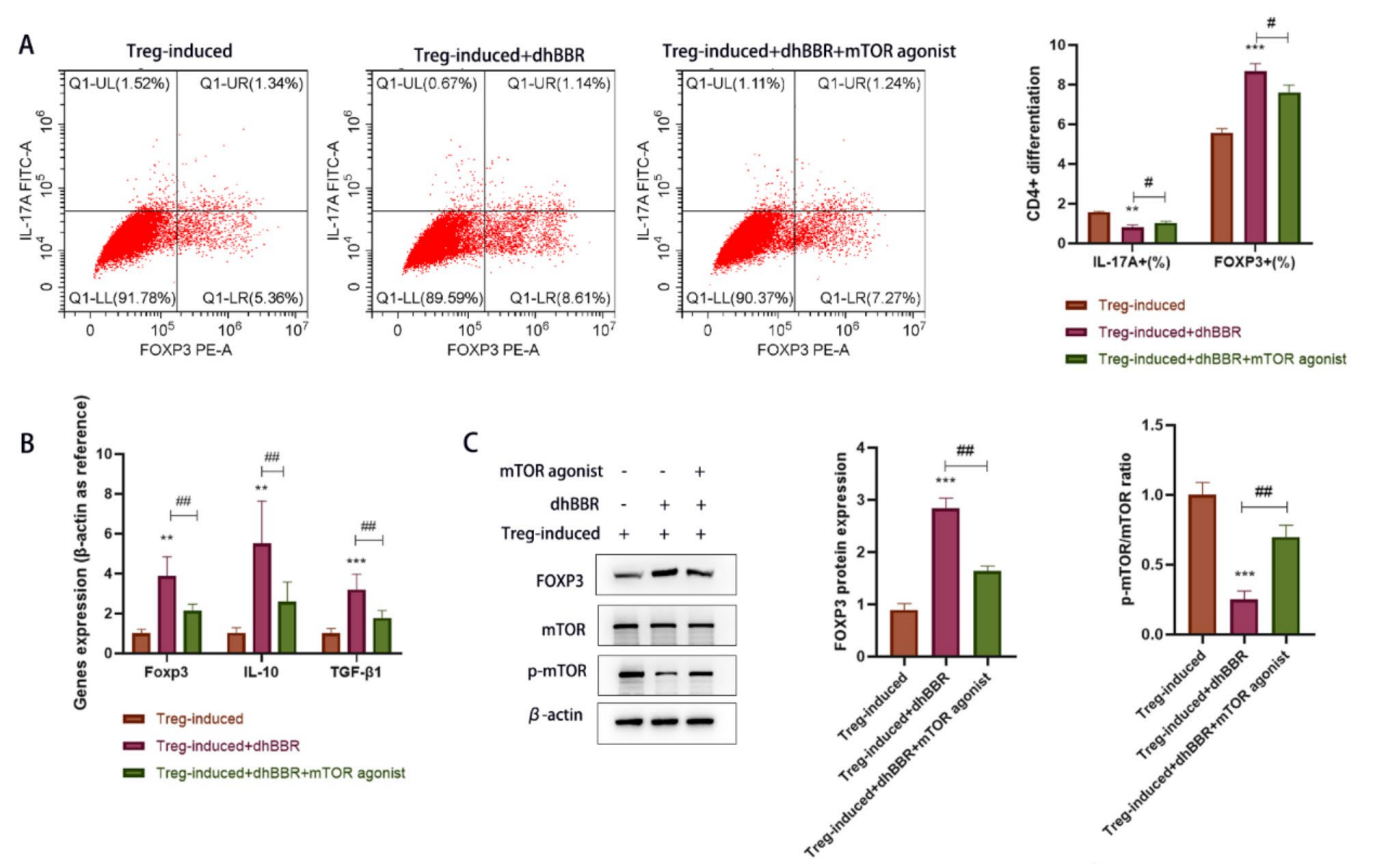
dhBBR inhibits CD4 + T cell differentiation into Tregs by inhibiting the mTOR signaling pathway. **A**: flow cytometry, the dual staining for FOXP3 and IL-17 A were used to identify Tregs and Th17 cells. **B**: qPCR was performed to quantify gene expressions of Foxp3, IL-10, and TGF-β1. **C**: proteins including mTOR, p-mTOR, and Foxp3 were detected by WB. vs. the Treg-induced group, ^**^p < 0.01, ^***^p < 0.001; vs. the Treg-induced + dhBBR group, ^#^*p* < 0.05, ^##^*p* < 0.01

MHY1485, an mTOR agonist, was used to validate the role of the mTOR pathway in dhBBR-mediated CD4 + T cell differentiation. As shown in Fig. 7A, MHY1485 reversed the dhBBR-induced decrease in IL-17 A + cells (%) and increase in FOXP3 + cells(%). As shown in Fig. 7B, Foxp3, IL-10, and TGF-β1 mRNA levels were decreased in the Treg + dhBBR + mTOR agonist group compared to those in the Treg + dhBBR agonist group. As shown in Fig. 7C, Foxp3 expression levels were similar among the three groups. p-mTOR/mTOR ratio was decreased by dhBBR and increased by MHY1485 treatment. These results suggest that dhBBR targets mTOR to promote CD4 + T cell differentiation into Treg cells.

### Role of the mTOR pathway in the inhibition of CD4 + T cell differentiation into Th17 cells by dhBBR

**Fig. 8:**
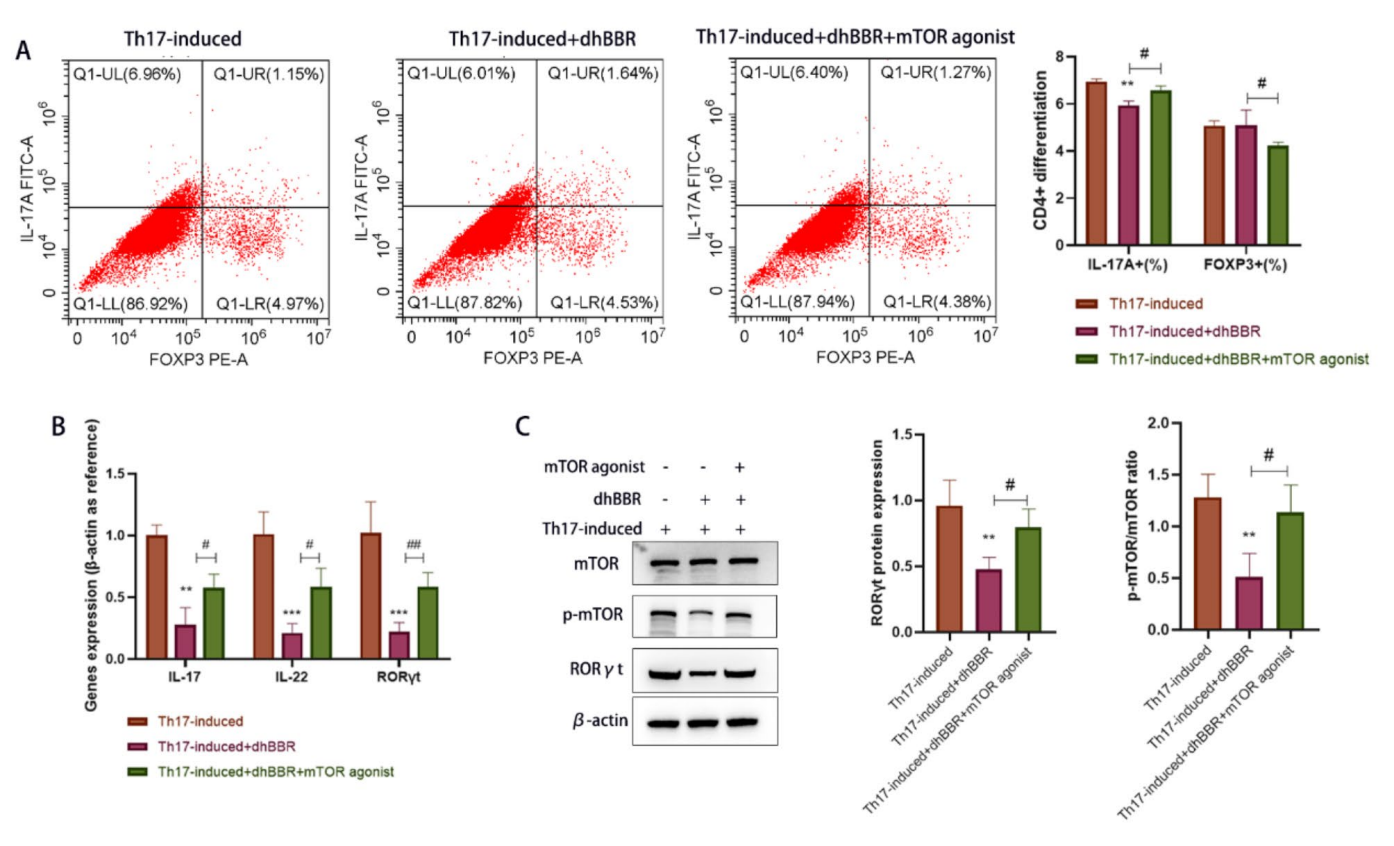
dhBBR inhibits CD4 + T cell differentiation into Th17 cells by inhibiting the mTOR signaling pathway. **A**: flow cytometry, the dual staining for FOXP3 and IL-17 A were used to identify Tregs and Th17 cells. **B**: qPCR was performed to quantify gene expressions of IL-17, IL-22, and RORγt. **C**: proteins including mTOR, p-mTOR, and RORγt were detected by WB. vs. the Th17-induced group, ^**^p < 0.01, ^***^p < 0.001; vs. the Th17-induced + dhBBR group, ^#^*p* < 0.05, ^##^*p* < 0.01

MHY1485 was used to treat the Th17-induced + dhBBR group and CD4 + T cells. As shown in Fig. 8A, MHY1485 treatment increased IL-17 A + cells(%) but decreased FOXP3 + cells(%) in the Th17-induced + dhBBR group. As shown in Fig. 8B, *IL-17*, *IL-22*, and *RORγt* mRNA levels were upregulated in the Treg + dhBBR + mTOR agonist group compared to those in the Treg + dhBBR agonist group. As shown in Fig. 8C, RORγt expression levels were similar among the three groups. Moreover, dhBBR decreased the p-mTOR/mTOR ratio, which was increased by MHY1485. These findings suggest that dhBBR targets mTOR to inhibit CD4 + T cell differentiation into Th17 cells.

## Discussion

In this study, we explored the therapeutic effects of dhBBR on POI model mice. Consistent with previous reports, this study showed that high-dose dhBBR reversed the POI-induced decrease in the number of follicles and ovarian mass. Sex hormones, including 17β-estradiol (E2), FSH, and AMH, are indicators of POI that collectively regulate the ovarian response. E2, which is derived from granulosa cells, is the primary estrogen maintaining the female reproductive system and secondary sexual characteristics. FSH is produced by the anterior pituitary gland, promotes the synthesis and secretion of E2, and facilitates follicle maturation (Medeiros et al. 2021). AMH originates from small antral follicles and reflects the ovarian follicle reserve in females (Shrikhande et al. 2020). Previous studies revealed that E2 and AMH levels are downregulated in mice with POI (Feng et al. 2024; Yang et al. 2024a, b), consistent with our results. Moreover, dhBBR improved the ovarian response in POI model mice. It also restored the Ki-67 and cleaved-caspase 3 levels to inhibit ovarian cell apoptosis.

Many genes playing important roles in the synthesis and regulation of sex hormones are associated with POI. Defects in Cyp17a1, Cyp19a1, and Cyp11a1 directly affect estrogen synthesis (Rojas et al. 2015). Star dysfunction adversely affects steroid hormone synthesis (Jefcoate et al. 2011). Abnormalities in Lhr affect ovulation and corpus luteum function (Timofeeva et al. 2024). However, in this study, qPCR analysis of POI model mice revealed significant downregulation of all these genes, but this effect was reversed by high doses of dhBBR. These findings suggest that dhBBR improves POI. However, the specific molecular mechanisms remain unclear, warranting further investigation.

POI is a reproductive disorder that is associated with autoimmunity(Torrealday et al. 2017; Szeliga et al. 2021). Th17 cells are CD4 + T cells primarily involved in the inflammatory and autoimmune responses (Lee et al. 2020). Th17 cell upregulation is commonly observed in POI patients (Gao et al. 2022). Therefore, an increase in Th17 cells could enhance the immune system’s attack on ovarian tissue. However, Treg cells, another form of CD4 + T cells, exerts an opposing effect by promoting the expression of TGF-β1, IL-10, and IL-35 (Yin et al. 2018). Recent POI studies have highlighted the potential of Th17 and Treg cells as biomarkers and therapeutic targets (Shuai et al. 2023; Liu et al. 2020). Therefore, investigating the specific mechanisms of Th17/Treg imbalance can aid in developing therapeutic methods to restore the immune balance, thereby delaying or reversing the progression of POI. In this study, dhBBR increased the Treg cell proportions but decreased the Th17 cell proportions. Moreover, rIL-17 and aCD25mAb confirmed that dhBBR restored the Th17/Treg cell balance to alleviate POI.

Th17/Treg cell differentiation is regulated by mTOR signaling(Liu et al. 2021; Shi et al. 2019). Activation of mTOR upregulates RORγt to promote Th17 cell differentiation and downregulates Foxp3 to inhibit Treg cell differentiation. Kailin Yang et al. used systems pharmacology and animal experiments to demonstrate that Gouqi Nuzhen Liuhe Decoction maintains ovarian homeostasis in POI model mice by inhibiting PI3K/mTOR pathway activation (Yang et al. 2024a, b). Variations in tuberous sclerosis complex genes affect the mTOR pathway and mediate the development of POI (Xu et al. 2022). Therefore, modulation of mTOR signaling is a potential target to restore the Th17/Treg cell balance in POI. Here, our animal and cell experiments demonstrated that dhBBR inhibited mTOR phosphorylation, similar to Treg induction. This suggests that dhBBR activates the mTOR pathway to mediate the Th17/Treg cell balance in POI model mice. However, the relationship between dhBBR and mTOR remains unclear.

Next, we explored the relationship between dhBBR and mTOR. Molecular docking analysis revealed Rheb as a target of dhBBR. Upon Rheb degradation, mTOR activity is significantly diminished, indicating that Rheb functions as an mTOR-activating transcription factor (Long et al. 2005). Rheb-GTP is the activated form of Rheb (Lu et al. 2020). In this study, Treg induction significantly decreased, whereas Th17 induction significantly increased Rheb levels in CD4 + T cells. dhBBR significantly decreased GTP-Rheb levels in Th17-induced CD4 + T cells. These findings suggest that dhBBR targets GTP-Rheb to regulate mTOR activation. Consequently, we conducted Co-IP experiments to confirm the relationship between dhBBR and Rheb/mTOR pathway. We also used an mTOR agonist for Th17 and Treg induction. Notably, dhBBR restored the Th17/Treg cell balance by inhibiting mTOR activation. In conclusion, this study showed that dhBBR targets the Rheb/mTOR pathway to promote CD4 + cell differentiation into Treg cells, thereby alleviating POI. However, some limitations are worth noting in the present study. The effects of dhBBR on clinical POI patients and the balance of Th17/Treg cells need to be further studied.

## Electronic supplementary material

Below is the link to the electronic supplementary material.

Supplementary Material 1

Supplementary Material 2

## Data Availability

No datasets were generated or analysed during the current study.

## Abbreviations

POI: Premature ovarian insufficiency
dhBBR: Dihydroberberine
E2: 17β-Estradiol
FSH: Follicle-stimulating hormone
BBR: Berberine
FSH: Follicle-stimulating hormone
AMH: Anti-Mullerian hormone
Star: Steroidogenic acute regulatory protein
Lhr: Luteinizing Hormone Receptor
GNLHD: Gouqi Nuzhen Liuhe Decoction
TSC: Tuberous sclerosis complex
Rheb: Ras homolog enriched in brain
mTORC1: The mammalian target of rapamycin complex 1
pZP3: Zona pellucida glycoprotein 3 peptide
FCM: Flow cytometry
ELISA: Enzyme-linked immunosorbent assay
Co-IP: Immunoprecipitation
IF: Immunofluorescence
