# Supplementary material for: Dihydroberberine alleviates Th17/Treg imbalance in premature ovarian insufficiency mice via inhibiting Rheb/mTOR signaling
Source: Mol Med. 2024 Oct 29;30:194. doi: 10.1186/s10020-024-00971-z (PMC11523677; doi:10.1186/s10020-024-00971-z)
Supplement: Supplementary file 1 — Supplementary Material 1 [file 10020_2024_971_MOESM1_ESM.pdf]

Wiley Editing Services

ENGLISH EDITING CERTIFICATE

This document certifies that the manuscript listed below was edited for proper English language, grammar, punctuation, spelling, and overall style by one or more of the highly qualified native English speaking editors at Wiley Editing Services

Manuscript title

Dihydroberberine restores the Th17/Treg cell balance in mice with premature ovarian insufficiency by inhibiting Rheb/mTOR signaling

Authors

Disi Deng1,2, Yeke Wu3, Keming Wu1, Nan Zeng2*, Wanjing Li4*

Order No

VJIYC_1

Date Issued

September 17, 2024

This document certifies that the manuscript listed above was edited for proper English language, grammar, punctuation, spelling, and overall style. Neither the research content nor the authors' intentions were altered in any way during the editing process. Documents receiving this certification should be English-ready for publication; however, the author has the ability to accept or reject our suggestions and changes. If you have any questions or concerns about this document or certification, please contact help-cn@wileyeditingservices.com.

Wiley Publishing Services is a service of Wiley Publishing. Wiley's Scientific, Technical, Medical, and Scholarly (STMS) business serves the world's research and scholarly communities, and is the largest publisher for professional and scholarly societies. Wiley is committed to providing high quality services for researchers. To find out more about Wiley Editing Services, visit http://wileyeditingservices.com. To learn more about our other author services provided by Wiley

WILEY

Publishing, visit https://authorservices.wiley.com/
